# The impact of past temporal discounting on mental health: Opposite effects of positive and negative event aftertastes over time: Aftertaste and time

**DOI:** 10.1016/j.ijchp.2024.100453

**Published:** 2024-03-03

**Authors:** Bowen Hu, Shunmin Zhang, Peiwei Liu, Feng Zhou, Tingyong Feng

**Affiliations:** a Faculty of Psychology, Southwest University, China; b Department of Psychology and Behavioral Sciences, Zhejiang University, China; c Department of Psychology, University of Florida, United States; d Key Laboratory of Cognition and Personality, Ministry of Education, Chongqing, China

**Keywords:** Temporal discounting, Mental health, Affect, Fading affect bias, Longitudinal tracking

## Abstract

**Background:**

Time frees people from bereavement, but also fades childhood happiness, these dynamics can be understood through the framework of past temporal discounting (PTD), which refers to the gradual decrease in affect intensity elicited by recalling positive or negative events over time. Despite its importance, measuring PTD has been challenging, and its impact on real-life outcomes, such as mental health remains unknown.

**Method:**

Here, we employed a longitudinal tracking approach to measure PTD in healthy participants (*N* = 210) across eight time points. We recorded changes in affect intensity for positive and negative events and examined the impact of PTD on mental health outcomes, including general mental well-being, depression, stress sensitivity, and etc.

**Results:**

The results of Bayesian multilevel modeling indicated that the affect intensity for positive and negative events discounted over time at a gradually decelerating rate. Furthermore, we found that maintaining good mental health heavily depended on rapid PTD of negative events and slow PTD of positive events.

**Conclusions:**

These results provide a comprehensive characterization PTD and demonstrate its importance in maintaining mental health.

Time, always flowing forward, drags us away from past events dispassionately, whether happy or sad. During this progression, the intensity of affect elicited by recalling past events (hereinafter referred to as “affect intensity for past events”) discounted as time goes by, being a past form of temporal discounting (Trope & Liberman, 2003, 2010), which is similar to how aftertastes of food gradually fade away over time. For example, the happiness of recalling a past vacation and the sorrow of recalling a failed relationship gradually faded across time. Past temporal discounting (PTD) is akin to the concept of future temporal discounting in the field of decision-making, where future reward (or loss) is perceived as less attractive (or aversive) as their delivery time delayed, or in the field's terminology, that future reward (or loss) has delay-discounted subjective value (Frederick et al., 2002; Kable & Glimcher, 2007).

Psychological mechanisms potentially underlying past temporal discounting and future temporal discounting have been suggested. The construal-level theory posits that temporally distant events, whether in the future or past, are psychologically distant and are evaluated with high levels of abstractness and reduced vividness (Trope & Liberman, 2010), resulting in discounted subjective value (Peters & Büchel, 2010) or discounted affect intensity (LaBar & Cabeza, 2006; Lin et al., 2016). Mental time travel theory suggests that PTD and future temporal discounting rely on the ability to “project oneself into the past and future,” underpinned by the neural network responsible for envisioning the future and remembering the past (Boyer, 2008; Buckner & Carroll, 2007; Schacter et al., 2012). Although there is well-established knowledge of future temporal discounting (Kable & Glimcher, 2007; Lempert et al., 2019; Peters & Büchel, 2010), the existence and effective observation of PTD still lack verification and cannot be straightforwardly extended from research on future temporal discounting.

This discrepancy arises from the fact that future temporal discounting fundamentally stems from the subjective valuation of hypothetical outcomes yet to be delivered in the future, in contrast, PTD fundamentally stems from the affect intensity for factual experiences that have already occurred in the past. Consequently, while future temporal discounting is primarily shaped by individuals' subjective perception of time (Zauberman et al., 2009), PTD is more likely to be influenced by the objective passage of time experienced in the real world. On the other hand, future temporal discounting is entangled with factors related to future time, such as uncertainty (Luhmann et al., 2008), anticipation (Iigaya et al., 2020), and waiting (Xu et al., 2020), while PTD is exempt from the influence of these factors. In summary, although there may be shared psychological mechanisms and neural substrates (Boyer, 2008; Buckner & Carroll, 2007; Schacter et al., 2012; Trope & Liberman, 2010), it is inappropriate to indiscriminately extend the findings and conclusions from research on future temporal discounting to PTD which was inadequately characterized with only several pieces of inappropriate (flawed in methodology) and indirect evidence.

Previous attempts to directly investigate PTD, were hindered by invalid approaches. Specifically, in these attempts, participants were forced to “choose” (Bickel et al., 2008; Pope et al., 2019; Yi et al., 2009) between two outcomes delivered in the past, which was devoid of practical meaning and was not relevant to real-world situations. Consequently, these attempts failed to provide credible evidence for PTD. Indirect evidence for PTD was also provided by cross-sectional comparison between the affect intensity for different events happened at different times, which demonstrated lower affect intensity for events that happened in more distant past (Gibbons et al., 2011; Holmes, 1970; Walker et al., 1997), but the credibility of which was limited since it cannot separate the confounding effects of events themselves and time on affect intensity (Skowronski et al., 2014). Meanwhile, PTD received indirect support from research on the development of posttraumatic stress symptoms (PTSS), in which the dynamic characteristics of the PTSS development (decreasing over time) in bereaved parents (Ljungman et al., 2015; Meert et al., 2011) and in survivors of terrorism (Birkeland et al., 2017) were effectively captured through longitudinal tracking. In summary, the endeavor made to investigate PTD had brought only unreliable evidence due to the flaws in research paradigms, which underscores the necessity to develop an effective measurement for PTD. Nonetheless, inspired by the research on the development of PTSS, longitudinal tracking stands out as a promising approach for effectively characterizing PTD.

Research of the PTSS development, furthermore, has implicated the potential significance of robust PTD paths in mental health. Robust PTD paths is defined as a pattern of slow discounting of the affect intensity for positive events and rapid discounting of the affect intensity for negative events (such as PTSS) over time, which serves to adaptively maintain mental health. The effects of robust PTD paths on mental health are partially supported by the research of fading affect bias (FAB). The FAB refers to the tendency that the affect intensity generally fades more rapidly for negative memories than positive memories in healthy individuals (Gibbons et al., 2011; Holmes, 1970; Lindeman et al., 2017; Walker et al., 1997), but not in depressed individuals (Walker et al., 2003; Walker & Skowronski, 2009). In the same vein, non-robust PTD paths (i.e., rapid discounting of the affect intensity for positive events or slow discounting of the affect intensity for negative events) could lead to diminished positive affect and amplified negative affect when recalling everyday past life events, which would become a risk factor for mental disorders. For instance, when recalling past negative events, patients with posttraumatic stress disorder or depression tended to frequently ruminate on them accompanied by amplified negative affect (Donaldson & Lam, 2004; Eisma et al., 2022; Nolen-Hoeksema et al., 2008), which might be explained by non-robust PTD paths for negative events (PTD-negative). On the other hand, when recalling past positive events, the patients with depression experienced less positive affect compared with the controls (Begovic et al., 2017), which might be explained by non-robust PTD paths for positive events (PTD-positive). Based on past research, robust PTD paths likely help maintain mental health, but non-robust PTD paths will increase the risk of mental disorders.

Research has found that PTD is a common experience in everyday life and it may potentially account for mental health. However, the absence of a valid research paradigm limits our understanding of the general characteristics of (robust) PTD and its specific impact on mental health. To validly capture the essence of PTD, unlike previous research using forced-choice paradigm (Bickel et al., 2008; Pope et al., 2019; Yi et al., 2009) or comparing different events (Gibbons et al., 2011; Holmes, 1970; Walker et al., 1997), we employed a longitudinal tracking approach to record changes in affect intensity for past positive and negative events across eight time points, and modeled the dynamic impact of time on affect intensity via Bayesian multilevel modeling. After characterizing the development of affect intensity for past positive and negative events over time at the population level, we quantified the discounting rates of PTD-positive and PTD-negative paths for each participant based on individual PTD-positive and PTD-negative paths. Finally, we assessed the impact of the discounting rates of PTD-positive and PTD-negative paths on multiple aspects of mental health with linear regression models.

## Methods

### Participants

Two hundred and forty college students were randomly recruited from a local university. Thirty participants were subsequently excluded from the analysis, with 28 participants failing to complete all tracking questionnaires and 2 participants displaying significantly abnormal discounting patterns compared with the majority of the sample (see supplementary material for details), leading to a final sample of 210 participants for further analyses (170 females; mean age 19.74 years, SD 1.36). This study was approved by the Institutional Review Board of the local university.

To estimate the power of the current sample for Bayesian multilevel modeling, a post-hoc power analysis was conducted via a Monte Carlo simulation in R with SIMR package (Green & MacLeod, 2016). Based on the effect size estimated from Bayesian multilevel modeling (see Table 1 for details), we found that the current sample size (*N* = 210) yielded 96.5 % (95 % CI, [95.17 %, 97.55 %]) and 93.8 % (95 % CI, [92.12 %, 95.21 %]) power to detect PTD-positive and PTD-negative paths with a significance level of 0.05, indicating that the sample was sufficient.

**Table 1:** Parameters and corresponding 95 % HPDI estimated by Bayesian multilevel modeling of the linear and quadratic effects of time on affect intensity for past positive and negative events.

Outcome variable	Parameter	Estimate	95 % HPDI	$\hat{R}$	ESS
Affect intensity for positive events	Intercept	67.806	[63.574, 72.047]	1.00	1982
	Time	−0.816	[−1.022, −0.605]	1.00	3286
	Time^2^	0.007	[0.004, 0.01]	1.00	3833
Affect intensity for negative events	Intercept	−51.569	[−55.824, −47.299]	1.00	4288
	Time	0.683	[0.502, 0.861]	1.00	6973
	Time^2^	−0.006	[−0.009, −0.003]	1.00	7293

*Note:* HPDI = highest posterior density interval. $\hat{R}$ = Gelman-Rubin's R-hat statistics. ESS = Bulk effective sample size.

### Measurements and procedure

Participants came to the laboratory and provided informed consent prior to completing a series of questionnaires. Then, the participants completed a questionnaire about past events, in which they recalled three positive events and three negative events that had happened to them in the past month (“Please recall a (un)happy event that had happened to you in the past month”). Next, participants were asked to name each event (“Please name this event”). They were also asked to provide a detailed description of what had happened (“Please describe this event succinctly without missing any important details”) and the exact date when the event occurred (“When did this event happen”), as well as to rate the intensity of their affective experience during each event on a 0 to 100 scale (“How were you feeling during this event?”, “0 ∼ 100, neutral – extremely (un)happy”). This rating should be at least 35 so recalling the event in the future could still arouse a detectable degree of affective experience for the participants. Otherwise participants were required to report another event up to the standard. Then, the names and description of the six events were saved to the note-taking app on participants’ mobile phones for further follow-ups. Finally, participants received payment for their participation, and they were encouraged to complete the 8 consecutive follow-ups (once every 4 days), which started 4 days later.

Participants received their first follow-up survey online 4 days after the laboratory interview. To control for the effect of memory vividness on affective experience when recalling past events (Lindeman et al., 2017; Sharot et al., 2004), identical descriptions of past events were provided across all of the follow-up surveys. Specifically, in the follow-up survey, participants were first instructed to retrieve the names and descriptions of each event from their note-taking app (“Please open the notes about the six past events recorded in your note-taking app, and copy their names and description to this survey”). After copying this information, participants were asked to rate their affective experience when recalling each past event on a −100 to 100 visual analog scale (VAS; “How does the event make you feel overall when you recall it now?”, “−100 ∼ 0 ∼ 100, extremely unhappy ∼ neutral ∼ extremely happy”), the ratings of which indicated the affect intensity elicited by recalling past events. A total of 8 follow-up surveys were delivered to each participant at 8 consecutive time points (once every four days). Afterward, the participants received extra payment for their participation. The participants had no explicit information about their valuations in the previous follow-ups, so that they could give unbiased valuations on their affective experience at each time point. For each participant, the affect intensity for 3 positive events and 3 negative events at 8 time points was obtained, and the time distance between each follow-up and the day that the event happened was calculated (ranged from 4 to 63 days).

After completing all follow up surveys, six questionnaires were adopted to assess mental health from multiple aspects. The Warwick-Edinburgh Mental Well-Being Scale (WEMWBS; Tennant et al., 2007) was used to assess general mental well-being, the Beck's Depression Inventory (BDI; Beck et al., 1988) was used to assess the severity of depression proneness, the Trait Anxiety Inventory (TAI; Spielberger et al., 1971) was used to assess anxiety proneness, the Perceived Stress Scale (Cohen et al., 1983) was used to assess stress sensitivity, and the Ruminative Responses Scale (RRS; Treynor et al., 2003) was used to assess rumination proneness. The total score of each scale was calculated and it was used to indicate the level of mental health.

### Bayesian estimation of the dynamic impact of time on affect intensity for past events

The PTD-positive and PTD-negative paths were characterized respectively with quadratic models. In regression models, the inclusion of the linear term can capture the directional trend of the affect intensity (dependent variable) with changes in time (independent variable), while extending the model by adding the quadratic term (second-order, quadratic model) allows for a more nuanced representation of the changing rate of the affect intensity as time varies. Specifically, we fit Bayesian mixed-effects quadratic models to the full sample (3 positive or negative events from 210 participants across 8 time points), in which nested random effects were incorporated into the models to account for the effects of events and participants (the event level was nested within the participant level), while the time distance (ranged from 4 to 63 days) and its quadratic term were incorporated as fixed effects. The modeling analysis was carried out with brms package (version 2.19.0; Bürkner, 2017) in R programming language (version 4.1.3; R Core Team, 2022), in which the posterior distribution was sampled by implementing a Markov Chain Monte Carlo simulation with four chains (4000 iterations each, with the 2000 first iterations used as warm-up samples).

Both models converged well, as indicated by all $\hat{R}$ value being close to 1 (< 1.01) and by high effective sample size (> 1000) and by a visual inspection of the trace plots (see Table 1 and Figure S1 in Supplementary Material; Gelman et al., 2015). The posterior predictive checks showed that the posterior predictive distributions generated by both models were similar to the distributions of observed data, indicating good fit to the data by both models (see Figure S2). Mixed-effects cubic models were also performed, but they experienced convergence problem ($\hat{R}$ value > 1.05) and were, therefore, discarded from further analysis.

### Quantifying rates of past temporal discounting for each individual

In the current study, we also aimed to quantify the rates of PTD-positive and PTD-negative paths for individuals. First, the random intercepts and random slopes for each individual were extracted from the mixed-effects models, which were used to characterize PTD-positive and PTD-negative paths for individuals. Based on the PTD-positive and PTD-negative individual paths, we then generated predicted outcomes for positive and negative affect intensity across a series of time distance (ranging from 4 to 63 days). Next, since the current study focused on the changing rate of affect intensity rather than the degree of affect intensity for past events, we scaled the time distance between 0 and 1 (by first subtracting 4, then dividing by 59), and scaled the predicted affect intensity by dividing by the first predicted affect intensity. Finally, the area under the curve (AUC) was calculated for the scaled PTD curve, formed by plotting the scaled predicted affect intensity (y-axis) against the scaled time distance (x-axis), which quantified the rates of PTD-positive (AUC-positive) and PTD-negative (AUC-negative) paths, respectively, for each individual (Harrison & McKay, 2012; Jimura et al., 2013; Shamosh et al., 2008). Notably, the values of AUC-negative for 2 participants were less than 0, which violated the definition of AUC. Therefore, these 2 participants were excluded from subsequent analysis.

### Statistical analysis

To investigate the relationship between AUC-positive and AUC-negative paths, Spearman's rank correlation was utilized to assess the correlation between the rates of PTD-positive and PTD-negative paths. And to explore the potential difference in the rates between PTD-positive and PTD-negative paths (implicated by "fading affect bias"; Lindeman et al., 2017; Marsh et al., 2019; Montijn et al., 2021; Walker et al., 1997), a paired sample *t*-test was implemented to examine the difference between AUC-positive and AUC-negative. Permutation test (1000 permutations) was used to determine the significance levels.

Next, the impact of PTD-positive and PTD-negative paths on mental health was explored by constructing multiple linear regression models, in which AUC-positive and AUC-negative were independent variables and each measurement of mental health was the dependent variable. We also examined the effect of PTD bias (the difference between normalized AUC-positive and normalized AUC-negative) on multiple aspects of mental health with Spearman's rank correlation.

### Transparency and openness

We report how we determined our sample size, all data exclusions, and all measures in the study. The analysis code for this study is publicly accessible from https://osf.io/9depy/?view_only=8d33580bb3a64cea9ee688b17f88f64a. The data used in the research is not available online due to ethical principles and the protection of the privacy of participants. The current study does not involve with any novel or unusual stimulus materials, all materials have been described in detail in the Methods section. There is not a preregistration for this study.

## Results

### Affect intensity for past events discounted over time at a gradually decelerating rate

**Fig. 1:**
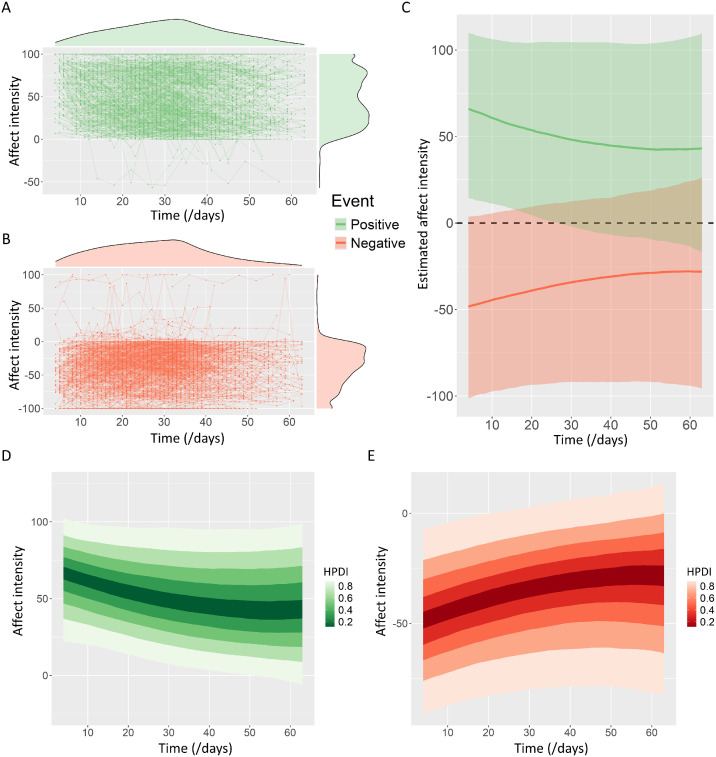
Affect intensity for positive and negative events discounts over time. Spagetti plots show the affect intensity for all of the past positive **(A)** and negative **(B)** events tracked across eight time points (630 positive events were colored in green, and 630 negative events were colored in red). The density plots at the margins of the spagetti plots show the distribution of time and affect intensity. **(C)** Estimated marginal means (lines) with their 95 % confidence interval (shades) of affect intensity across time for past positive (colored in green) and negative (colored in red) events, indicating the marginal effect of time on affect intensity. Distribution plots of posterior predicted means for affect intensity for past positive **(D)** and negative **(E)** events. The posterior predicted means are generated with new/unobserved participants. Deeper color (lower HPDI) indicates that the values of posterior predicted means are more certain. HPDI = highest posterior density interval.

Fig. 1A and B show the affect intensity for all positive and negative events tracked across eight time points. The results from Bayesian multilevel modeling showed significant (indicated by the 95 % highest posterior density intervals that did not contain zero) linear and quadratic effects of time on affect intensity for both positive (linear effect: *B* = −0.816, 95 % HPDI = [−1.022, −0.605]; quadratic effect: *B* = 0.007, 95 % HPDI = [0.004, 0.01]) and negative (linear effect: *B* = 0.683, 95 % HPDI = [0.502, 0.861]; quadratic effect: *B* = −0.006, 95 % HPDI = [−0.009, −0.003]) events at the population level, which were summarized in Table 1 and Figure S3. Fig. 1C shows the population-level effects of time on affect intensity for both positive (colored in green) and negative (colored in red) events by estimating the marginal effect of time on affect intensity, which showed that overall, the affect intensity decreased over time at a gradually decelerating rate for both of the positive and negative events. The significant quadratic effect and the curved lines both indicated that the discounting rates gradually decreased over time. The distribution of posterior predicted means for unobserved participants based on Bayesian multilevel modeling were displayed in Fig. 1D and E, which also showed a discounting pattern similar to the one revealed by the marginal effect results in Fig. 1C. These results suggested a general rule for both PTD-positive and PTD-negative, that the affect intensity for both positive and negative events discounted over time at a gradually decelerating rate.

### Association and difference between the rates of PTD-positive and PTD-negative paths

**Fig. 2:**
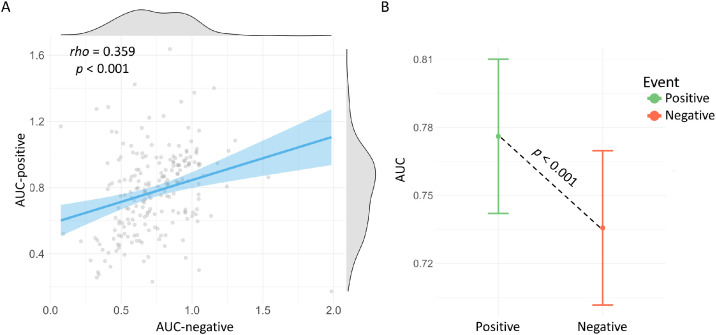
**(A)** Scatter plot indicates the correlation between AUC-positive and AUC-negative, with blue line and shade representing the linear fit line and its 95 % confidence interval. The density plots at the margins of the scatter plot show the distribution of AUC-positive and AUC-negative. **(B)** Mean plot indicates the difference between the means of AUC-positive (colored in gree) and AUC-negative (colored in red). The *p-*values were calculated via permutation tests.

We further conducted an exploratory analysis on the association and difference between the discounting rates of past positive and negative events, based on AUC-positive and AUC-negative, which were estimated for each individual as indicators of discounting rates. We found a significant positive correlation between AUC-positive and AUC-negative, Spearman's *rho* = 0.359, *permu-p* < .001, see Fig. 2A. And AUC-positive (*M* = 0.776, *SD* = 0.249) was significantly higher than AUC-negative (*M* = 0.736, *SD* = 0.258), *t* = 1.93, *permu-p* < .001, Cohen's *d* = 0.134, see Fig. 2B. The results indicate that individuals discounted past positive and negative events in a similar way, but that individuals discounted past negative events at a more rapid rate than past positive events.

### Multiple aspects of mental health were predicted by rates of PTD-positive and PTD-negative paths

**Fig. 3:**
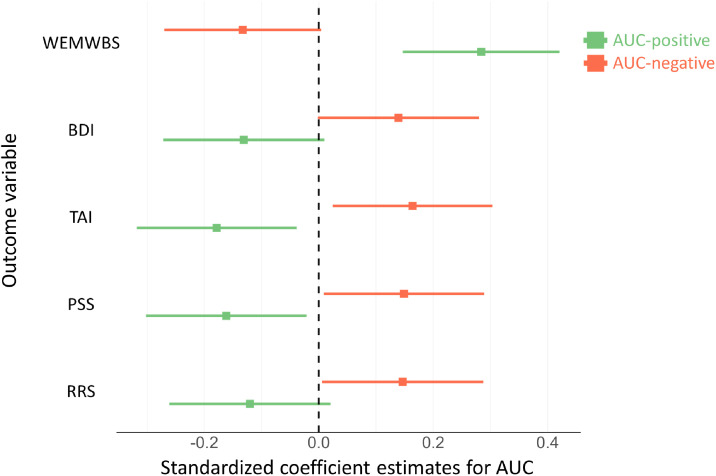
Dot-and-whisker plot shows the standardized coefficient estimates (dots) and corresponding 95 % confidence intervals (whiskers) of multiple regression models which predict multiple aspects of mental health by AUC-positive (colored in green) and AUC-negative (colored in red). WEMWBS = Warwick-Edinburgh Mental Well-Being Scale. BDI = Beck's Depression Inventory. TAI = Trait Anxiety Inventory. PSS = Perceived Stress Scale. RRS = Ruminative Responses Scale.

Using multiple linear regression models, we evaluated the effects of rates of PTD-positive (AUC-positive) and rates of PTD-negative (AUC-negative) paths on multiple aspects of mental health. The results indicated that AUC-positive and AUC-negative showed significant or marginally significant effects on multiple aspects of mental health (see in Fig. 3; summarized in Table S1), including general mental well-being (AUC-positive: *β* = 0. 284, 95 % CI = [0.147, 0.421], *p* < .001; AUC-negative: *β* = −0.133, 95 % CI = [−0.27, 0.004], *p* = .057), depression proneness (AUC-positive: *β* = −0.131, 95 % CI = [−0.272, 0.01], *p* = .068; AUC-negative: *β* = 0.139, 95 % CI = [−0.002, 0.28], *p* = .053), anxiety proneness (AUC-positive: *β* = −0.178, 95 % CI = [−0.318, −0.039], *p* = .013; AUC-negative: *β* = 0.164, 95 % CI = [0.024, 0.303], *p* = .022), stress sensitivity (AUC-positive: *β* = −0.162, 95 % CI = [−0.302, −0.021], *p* = .024; AUC-negative: *β* = 0.149, 95 % CI = [0.009, 0.289], *p* = .038), rumination proneness (AUC-positive: *β* = −0.12, 95 % CI = [−0.261, 0.02], *p* = .093; AUC-negative: *β* = 0.147, 95 % CI = [0.006, 0.287], *p* = .042). Overall, multiple aspects of mental health would be globally impaired by rapid PTD-positive and slow PTD-negative paths.

We then examined the effects of PTD bias (the difference between normalized AUC-positive and normalized AUC-negative) on multiple aspects of mental health, by using Spearman's rank correlation. The results (see Figure S4 for scatter plots of the association) showed that PTD bias had significant correlations with general mental well-being (*rho* = 0.292, *p* < .001), depression proneness (*rho* = −0.16, *p* = .021), anxiety proneness (*rho* = −0.166, *p* = .017), stress sensitivity (*rho* = −0.235, *p* < .001), and rumination proneness (*rho* = −0.156, *p* = .025). The results indicated that individuals who discounted past negative events at a more rapid rate than positive events tended to have better mental health.

## Discussion

The dynamic impact of time on the affect intensity elicited by recalling past events, namely past temporal discounting (PTD), has not been adequately investigated and characterized with a valid research paradigm. Consequently, our understanding of its impact on mental health remains limited. Here, by employing a longitudinal tracking approach, we showed that the affect intensity for positive and negative events discounted over time at a gradually decelerating rate (well-described by the quadratic model), wherein the affect intensity for negative events discounted more rapidly. Furthermore, we found that rapid PTD paths of positive events and slow PTD paths of negative events consistently had an adverse effect on multiple aspects of mental health. Together, our findings provide a foundational understanding of PTD and reveal its comprehensive impact on mental health.

Previous research attempted to investigate PTD mainly through cross-sectional comparison between different events happened at different time (Gibbons et al., 2011; Holmes, 1970; Walker et al., 1997), which confounded the effect of time and events themselves on affect intensity (Skowronski et al., 2014), or through forcing participants to “choose” (Bickel et al., 2008; Pope et al., 2019; Yi et al., 2009) between two outcomes delivered in the past, which created an unrealistic scenario for the participants, thus both approaches failed to provide credible evidence. Inspired by PTSS development research (Ljungman et al., 2015; Meert et al., 2011), we recorded changes in affect intensity for past events via a longitudinal tracking approach, which helped to validly capture the general characteristics of PTD.

We found that the affect intensity for positive and negative events discounted over time at a gradually decelerating rate, similarly to the hyperbolic pattern of future temporal discounting, which was rooted in the valuation system (discounted subjective value was tracked by its neural response; Kable & Glimcher, 2007). Notably, the brain regions which formed this valuation system, namely the striatum and medial prefrontal cortex, were also strongly involved in affective reactions that would be integrated into subjective valuation (Phelps et al., 2014). This finding further implicates shared neural mechanisms underlying the overlapping characteristics observed in both PTD and future temporal discounting. Therefore, building upon the sound methodology and pioneering knowledge provided by the current study, future studies could explore the association between PTD and future temporal discounting along with the distinct and shared cognitive and neural mechanisms between them, eventually construct an integrated model that spans both past and future to enhance our understanding of the temporal effects (both objective passage of time and subjective perception of time) on the human mind.

Our finding that individuals generally discounted past negative events at a more rapid rate than past positive events, on the one hand, replicated the findings of fading affect bias research (FAB; Gibbons et al., 2011; Holmes, 1970; Lindeman et al., 2017; Walker et al., 1997) and extend the FAB research by providing causal evidence through longitudinal tracking. On the other hand, our findings were not adequately accounted for by the existing theories about the origin of FAB. Specifically, the FAB were considered to emerge from the bias in fading memory vividness (Gibbons et al., 2022; Lindeman et al., 2017; Ritchie & Batteson, 2013), which aligned with the research about actively forgetting unwanted (usually negatively valenced) memories (Anderson & Hanslmayr, 2014; Anderson & Hulbert, 2021). However, in the current study, despite the fact that the level of memory vividness was kept constant throughout the tracking period, affect intensity for negative events persisted to fade more rapidly than positive events. Therefore, our findings highlight an endogenous difference in the rates between PTD-positive and PTD-negative paths, which may not be exogenously attributed to the bias in fading memory vividness and may have independently contributed to FAB. Further exploration is necessary to understand the cognitive mechanisms and neural substrates underlying this endogenous difference (e.g., rooted in affective reactivity system) and its association with FAB and active forgetting.

Meanwhile, additional theories and hypotheses associated with FAB research, such as the mobilization-minimization hypothesis and self-enhancement theory, could account for the observed differences in the rates of PTD-positive and PTD-negative paths. According to the mobilization-minimization hypothesis, individuals mobilize physiological, cognitive, and behavioral responses to minimize the impact of negative events, thus promoting resilience and preserving positive self-regard (Taylor, 1991). Through this proactive process, the adverse consequences of past negative events (i.e., the negative emotions elicited upon recall) are rapidly discounted, resulting in a lower rate for PTD-negative paths compared to PTD-positive paths. According to another pivotal theory regarding the FAB, self-enhancement theory, the higher rates of PTD-positive paths compared with PTD-negative paths can be viewed as an effective psychological mechanism that enhances the positivity of self through biased memory processes (Sedikides & Skowronski, 2020). Therefore, future studies ought to further investigate the influence of various kinds of coping strategies (Kross et al., 2009) and top-down regulation (Engen & Anderson, 2018) on PTD.

Furthermore, our findings directly revealed the impacts of rates of PTD-positive and PTD-negative paths on multiple aspects of mental health. These findings highlight the crucial role of robust PTD paths as a fundamental mechanism in adaptively maintaining mental health through regulating everyday affect, suggesting its comparable adaptive value to the active forgetting (Anderson & Hulbert, 2021; Nørby, 2015) or to FAB (Ritchie et al., 2015; Sedikides & Skowronski, 2020). Specifically, we found that mental health of individuals benefited from robust PTD paths, which referred to rapid discounting of the affect intensity for negative events and slow discounting of the affect intensity for positive events over time. Robust PTD-positive paths (slow discounting of the affect intensity for positive events) would increase positive affect (“sweet aftertaste”) when recalling positive events and improve mental health, similarly to nostalgia (Layous et al., 2022). In contrast, non-robust PTD-negative paths (slow discounting of the affect intensity for negative events) would increase negative affect (“bitter aftertaste”) when recalling negative events and impair mental health, similarly to rumination (Moberly & Watkins, 2008). More importantly, considering that PTD-related psychological mechanisms, such as future temporal discounting (Lempert et al., 2019) and rumination (Ehring & Watkins, 2008), have been considered as transdiagnostic processes in mental disorders, the current findings may gain new perspectives and provide intriguing insights into psychiatry research. For example, from the PTD-based perspective, rumination in depression may emerge from a non-robust PTD-negative paths (temporally stable, not discounting over time) which is maladaptive as opposed to robust PTD-negative paths. This research can help researchers understand the dynamic mechanisms of rumination and improve the diagnosis precision of related mental disorders (Insel, 2014). On this foundation, clinicians can manage to predict the response to treatment approaches in patients and identify the treatment approaches that are best suited for patients based on their PTD characteristics (rates of PTD paths), such as whether patients are impacted by rapid PTD-positive paths, slow PTD-negative paths, or both. Specifically, patients with rapid PTD-positive paths are more likely to benefit from positive memory intervention and gain more experience of positive emotional states (Miguel-Alvaro et al., 2021), while patients with slow PTD-negative paths are more likely to benefit from the therapy focusing on negative memory, such as narrative exposure therapy (Bichescu et al., 2007).

These findings are limited to the population and time scale investigated in this study. To fully understand how non-robust PTD paths impairs mental health, future research still needs to characterize the pattern of PTD in special population (such as patients with mental disorders and trauma victims) and in larger time scales (such as months and years, as in PTSS research: Birkeland et al., 2017; Ljungman et al., 2015; Meert et al., 2011). Meanwhile, it is also essential to explore the extent to which closely associated factors like self-regulatory mechanisms contribute to PTD and how they operate within it, as suggested by research and theories related to FAB (Engen & Anderson, 2018; Sedikides & Skowronski, 2020).

In conclusion, our study provides crucial insights into the dynamic impact of past temporal discounting (PTD) on affect intensity and its implications for mental health. Through longitudinal tracking, we demonstrated that PTD affects the intensity of both positive and negative events, with negative events fading more rapidly over time. Rapid PTD paths of positive events and slow PTD paths of negative events consistently correlated with adverse effects on mental health. These findings underscore the significance of PTD in understanding and addressing mental health challenges. Further research exploring the cognitive mechanisms and neural substrates underlying PTD, along with its association with phenomena like fading affect bias and active forgetting, could contribute to a more comprehensive understanding of temporal effects on psychological well-being.

## Declaration of competing interest

The authors declare that they have no known competing financial interests or personal relationships that could have appeared to influence the work reported in this paper.

## Appendix Supplementary materials

Image, application 1

## References

[bib0001] Anderson M.C., Hanslmayr S. (2014). Neural mechanisms of motivated forgetting. Trends in Cognitive Sciences.

[bib0002] Anderson M.C., Hulbert J.C. (2021). Active forgetting: Adaptation of memory by prefrontal control. Annual Review of Psychology.

[bib0003] Beck A.T., Steer R.A., Carbin M.G. (1988). Psychometric properties of the beck depression inventory: Twenty-five years of evaluation. Clinical Psychology Review.

[bib0004] Begovic E., Panaite V., Bylsma L.M., George C., Kovacs M., Yaroslavsky I., Baji I., Benák I., Dochnal R., Kiss E., Vetró Á., Kapornai K., Rottenberg J. (2017). Positive autobiographical memory deficits in youth with depression histories and their never-depressed siblings. British Journal of Clinical Psychology.

[bib0005] Bichescu D., Neuner F., Schauer M., Elbert T. (2007). Narrative exposure therapy for political imprisonment-related chronic posttraumatic stress disorder and depression. Behaviour Research and Therapy.

[bib0006] Bickel W.K., Yi R., Kowal B.P., Gatchalian K.M. (2008). Cigarette smokers discount past and future rewards symmetrically and more than controls: Is discounting a measure of impulsivity?. Drug and Alcohol Dependence.

[bib0007] Birkeland M.S., Hansen M.B., Blix I., Solberg Ø., Heir T. (2017). For Whom does time heal wounds? Individual differences in stability and change in posttraumatic stress after the 2011 Oslo bombing. Journal of Traumatic Stress.

[bib0008] Boyer P. (2008). Evolutionary economics of mental time travel?. Trends in Cognitive Sciences.

[bib0009] Buckner R.L., Carroll D.C. (2007). Self-projection and the brain. Trends in Cognitive Sciences.

[bib0010] Bürkner P.-C. (2017). brms: An R package for Bayesian multilevel models using Stan. Journal of Statistical Software.

[bib0011] Cohen S., Kamarck T., Mermelstein R. (1983). A global measure of perceived stress. Journal of Health and Social Behavior.

[bib0012] Donaldson C., Lam D. (2004). Rumination, mood and social problem-solving in major depression. Psychological Medicine.

[bib0013] Ehring T., Watkins E.R. (2008). Repetitive negative thinking as a transdiagnostic process. International Journal of Cognitive Therapy.

[bib0014] Eisma M.C., Franzen M., Paauw M., Bleeker A., aan het Rot M. (2022). Rumination, worry and negative and positive affect in prolonged grief: A daily diary study. Clinical Psychology & Psychotherapy.

[bib0015] Engen H.G., Anderson M.C. (2018). Memory control: A fundamental mechanism of emotion regulation. Trends in Cognitive Sciences.

[bib0016] Frederick S., Loewenstein G., O'donoghue T (2002). Time discounting and time preference: A critical review. Journal of Economic Literature.

[bib0017] Gelman A., Carlin J.B., Stern H.S., Dunson D.B., Vehtari A., Rubin D.B. (2015).

[bib0018] Gibbons J.A., Dunlap S., Friedmann E., Dayton C., Rocha G. (2022). The fading affect bias is disrupted by false memories in two diary studies of social media events. Applied Cognitive Psychology.

[bib0019] Gibbons J.A., Lee S.A., Walker W.R. (2011). The fading affect bias begins within 12 hours and persists for 3 months. Applied Cognitive Psychology.

[bib0020] Green P., MacLeod C.J. (2016). SIMR: An R package for power analysis of generalized linear mixed models by simulation. Methods in Ecology and Evolution.

[bib0021] Harrison J., McKay R. (2012). Delay discounting rates are temporally stable in an equivalent present value procedure using theoretical and area under the curve analyses. The Psychological Record.

[bib0022] Holmes D.S. (1970). Differential change in affective intensity and the forgetting of unpleasant personal experiences. Journal of Personality and Social Psychology.

[bib0023] Iigaya K., Hauser T.U., Kurth-Nelson Z., O'Doherty J.P., Dayan P., Dolan R.J (2020). The value of what's to come: Neural mechanisms coupling prediction error and the utility of anticipation. Science Advances.

[bib0024] Insel T.R. (2014). The NIMH Research Domain Criteria (RDoC) project: Precision medicine for psychiatry. American Journal of Psychiatry.

[bib0025] Jimura K., Chushak M.S., Braver T.S. (2013). Impulsivity and self-control during intertemporal decision making linked to the neural dynamics of reward value representation. The Journal of Neuroscience.

[bib0026] Kable J.W., Glimcher P.W. (2007). The neural correlates of subjective value during intertemporal choice. Nature Neuroscience.

[bib0027] Kross E., Davidson M., Weber J., Ochsner K. (2009). Coping with emotions past: The neural bases of regulating affect associated with negative autobiographical memories. Biological Psychiatry.

[bib0028] LaBar K.S., Cabeza R. (2006). Cognitive neuroscience of emotional memory. Nature Reviews Neuroscience.

[bib0029] Layous K., Kurtz J.L., Wildschut T., Sedikides C. (2022). The effect of a multi-week nostalgia intervention on well-being: Mechanisms and moderation. Emotion (Washington, D.C.).

[bib0030] Lempert K.M., Steinglass J.E., Pinto A., Kable J.W., Simpson H.B. (2019). Can delay discounting deliver on the promise of RDoC?. Psychological Medicine.

[bib0031] Lin W.-J., Horner A.J., Burgess N. (2016). Ventromedial prefrontal cortex, adding value to autobiographical memories. Scientific Reports.

[bib0032] Lindeman M.I., Zengel B., Skowronski J.J. (2017). An exploration of the relationship among valence, fading affect, rehearsal frequency, and memory vividness for past personal events. Memory (Hove, England).

[bib0033] Ljungman L., Hovén E., Ljungman G., Cernvall M., von Essen L. (2015). Does time heal all wounds? A longitudinal study of the development of posttraumatic stress symptoms in parents of survivors of childhood cancer and bereaved parents. Psycho-Oncology.

[bib0034] Luhmann C.C., Chun M.M., Yi D.-J., Lee D., Wang X.-J. (2008). Neural dissociation of delay and uncertainty in intertemporal choice. Journal of Neuroscience.

[bib0035] Marsh C., Hammond M.D., Crawford M.T. (2019). Thinking about negative life events as a mediator between depression and fading affect bias. PloS one.

[bib0036] Meert K.L., Shear K., Newth C.J., Harrison R., Berger J., Zimmerman J., Anand K.J.S., Carcillo J., Donaldson A.E., Dean J.M. (2011). Follow-up study of complicated grief among parents eighteen months after a child's death in the pediatric intensive care unit. Journal of Palliative Medicine.

[bib0037] Miguel-Alvaro A., Guillén A.I., Contractor A.A., Crespo M. (2021). Positive memory intervention techniques: A scoping review. Memory (Hove, England).

[bib0038] Moberly N.J., Watkins E.R. (2008). Ruminative self-focus and negative affect. Journal of Abnormal Psychology.

[bib0039] Montijn N.D., Gerritsen L., Engelhard I.M. (2021). Forgetting the future: Emotion improves memory for imagined future events in healthy individuals but not individuals with anxiety. Psychological Science.

[bib0040] Nolen-Hoeksema S., Wisco B.E., Lyubomirsky S. (2008). Rethinking rumination. Perspectives on Psychological Science.

[bib0041] Nørby S. (2015). Why forget? On the adaptive value of memory loss. Perspectives on Psychological Science.

[bib0042] Peters J., Büchel C. (2010). Episodic future thinking reduces reward delay discounting through an enhancement of prefrontal-mediotemporal interactions. Neuron.

[bib0043] Phelps E.A., Lempert K.M., Sokol-Hessner P. (2014). Emotion and decision making: Multiple modulatory neural circuits. Annual Review of Neuroscience.

[bib0044] Pope D.A., Poe L., Stein J.S., Snider S.E., Bianco A.G., Bickel W.K. (2019). Past and future preference reversals are predicted by delay discounting in smokers and non-smokers. Experimental and Clinical Psychopharmacology.

[bib0045] R Core Team (2022). https://www.R-project.org/.

[bib0046] Ritchie T.D., Batteson T.J. (2013). Perceived changes in ordinary autobiographical events’ affect and visual imagery colorfulness. Consciousness and Cognition.

[bib0047] Ritchie T.D., Batteson T.J., Bohn A., Crawford M.T., Ferguson G.V., Schrauf R.W., Walker W.R. (2015). A pancultural perspective on the fading affect bias in autobiographical memory. Memory (Hove, England).

[bib0048] Schacter D.L., Addis D.R., Hassabis D., Martin V.C., Spreng R.N., Szpunar K.K. (2012). The future of memory: Remembering, imagining, and the brain. Neuron.

[bib0049] Sedikides C., Skowronski J.J. (2020). In human memory, good can be stronger than bad. Current Directions in Psychological Science.

[bib0050] Shamosh N.A., DeYoung C.G., Green A.E., Reis D.L., Johnson M.R., Conway A.R.A., Engle R.W., Braver T.S., Gray J.R. (2008). Individual differences in delay discounting: Relation to intelligence, working memory, and anterior prefrontal cortex. Psychological Science.

[bib0051] Sharot T., Delgado M.R., Phelps E.A. (2004). How emotion enhances the feeling of remembering. Nature Neuroscience.

[bib0052] Skowronski J.J., Walker W.R., Henderson D.X., Bond G.D., Olson J.M., Zanna M.P. (2014). Advances in experimental social psychology.

[bib0053] Spielberger C.D., Gonzalez-Reigosa F., Martinez-Urrutia A., Natalicio L.F., Natalicio D.S. (1971). The state-trait anxiety inventory. Revista Interamericana de Psicologia/Interamerican Journal of Psychology.

[bib0054] Taylor S.E. (1991). Asymmetrical effects of positive and negative events: The mobilization-minimization hypothesis. Psychological Bulletin.

[bib0055] Tennant R., Hiller L., Fishwick R., Platt S., Joseph S., Weich S., Parkinson J., Secker J., Stewart-Brown S. (2007). The Warwick-Edinburgh mental well-being scale (WEMWBS): Development and UK validation. Health and Quality of Life Outcomes.

[bib0056] Treynor W., Gonzalez R., Nolen-Hoeksema S. (2003). Rumination reconsidered: A psychometric analysis. Cognitive Therapy and Research.

[bib0057] Trope Y., Liberman N. (2003). Temporal construal. Psychological Review.

[bib0058] Trope Y., Liberman N. (2010). Construal-level theory of psychological distance. Psychological Review.

[bib0059] Walker W.R., Skowronski J., Gibbons J., Vogl R., Thompson C. (2003). On the emotions that accompany autobiographical memories: Dysphoria disrupts the fading affect bias. Cognition and Emotion.

[bib0060] Walker W.R., Skowronski J.J. (2009). The Fading affect bias: But what the hell is it for?. Applied Cognitive Psychology.

[bib0061] Walker W.R., Vogl R.J., Thompson C.P. (1997). Autobiographical memory: Unpleasantness fades faster than pleasantness over time. Applied Cognitive Psychology.

[bib0062] Xu P., González-Vallejo C., Vincent B.T. (2020). Waiting in intertemporal choice tasks affects discounting and subjective time perception. Journal of Experimental Psychology: General.

[bib0063] Yi R., Landes R.D., Bickel W.K. (2009). Novel models of intertemporal valuation: Past and future outcomes. Journal of Neuroscience, Psychology, and Economics.

[bib0064] Zauberman G., Kim B.K., Malkoc S.A., Bettman J.R. (2009). Discounting time and time discounting: Subjective time perception and intertemporal preferences. Journal of Marketing Research.

