# Supplementary material for: The impact of past temporal discounting on mental health: Opposite effects of positive and negative event aftertastes over time: Aftertaste and time
Source: Int J Clin Health Psychol. 2024 Mar 3;24(2):100453. doi: 10.1016/j.ijchp.2024.100453 (PMC10915560; doi:10.1016/j.ijchp.2024.100453)
Supplement: Supplementary file 1 [file mmc1.docx]

**Supplementary Material for**

The Impact of Past Temporal Discounting on Mental Health: Opposite Effects of Positive and Negative Event Aftertastes Over Time

**Discriminating abnormal discounting pattern among participants**

Before formal analysis, it was important to perform data cleaning to remove incorrect data in the dataset. In the current study, participants with abnormal discounting pattern were deemed as incorrect data and needed to be removed. These participants were operationally defined with an obviously deviant discounting rate, that they discounted past events at a significantly different speed from the majority (outside 3 standard deviations of the mean). In the current study, the discounting rates were not calculated until we completed Bayesian multilevel modeling and extracted the random intercepts and random slopes for each individual from the mixed-effect models to characterize individual paths of positive and negative PTD. However, including the participants with abnormal discounting pattern in the modeling would impair the convergence of modeling. Therefore, in order to detect and exclude these participants before modeling, we calculated a “rough” estimate of the discounting rates by using the original data instead of the estimated data from modeling.

First, tracking time points (ranged between 1 to 8), instead of days, were treated as time units. Therefore, for each individual, affect intensity for each event at each tracking time point was scaled by dividing by the affect intensity at first time point, and was then averaged across three positive events and three negative events respectively. Time was also scaled between 0 to 1 (by first subtracting 1, then dividing by 7). Finally, the averaged scaled affect intensity (y-axis) was plotted against the scaled time (x-axis) to form the scaled positive and negative PTD curves for each individual, and the area under the curve (AUC-rough) for the scaled PTD curves was calculated. The positive and negative AUC-rough of two participants (participant 1: positive AUC-rough = 6.798, negative AUC-rough = 3.758; participant 2: positive AUC-rough = 9.676, negative AUC-rough = 0.071) lied outside 3 standard deviations of the mean (N = 212; positive AUC-rough, M ± SD = 0.872 ± 0.78; negative AUC-rough, M ± SD = 0.824 ± 0.394), and was excluded from further analysis.

**Figure S1. Overlapping trace plots indicating good convergence and mixing of Markov Chain Monte Carlo simulation in models of positive PTD (A) and negative PTD (B)**


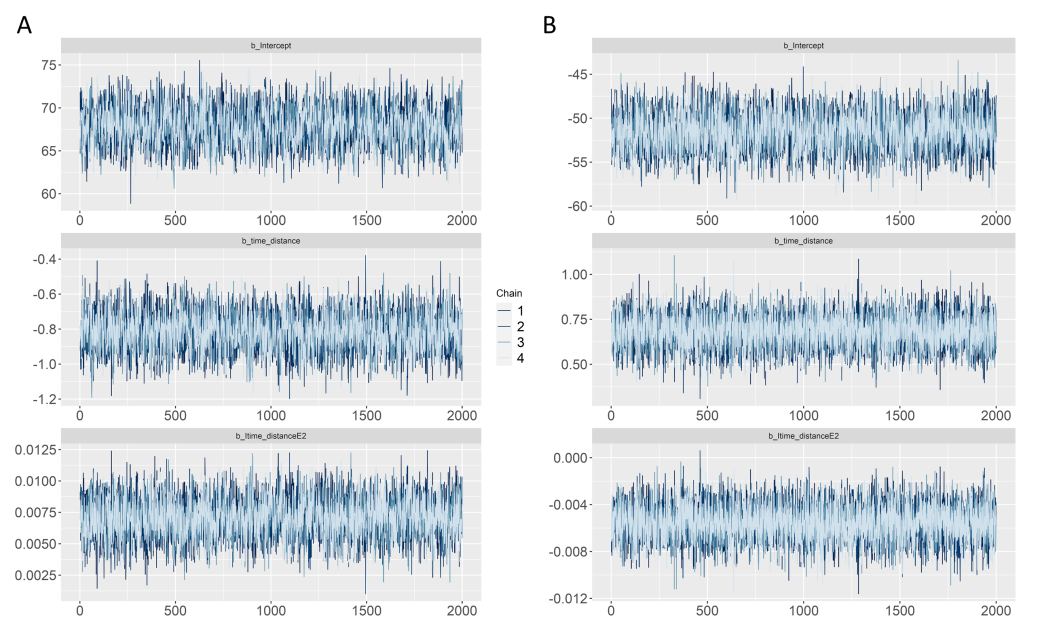


*Note.* The first row contains the trace plots for the intercepts, while the second and third rows contain the trace plots for linear and quadratic term of time distance. Of each trace plot, the y-axis shows the parameter values, while the x-axis shows the iteration number.

**Figure S2. Posterior predictive checks for assessment of the outcome (affective intensity) indicating good fit to the data by models of positive PTD (A) and negative PTD (B)**


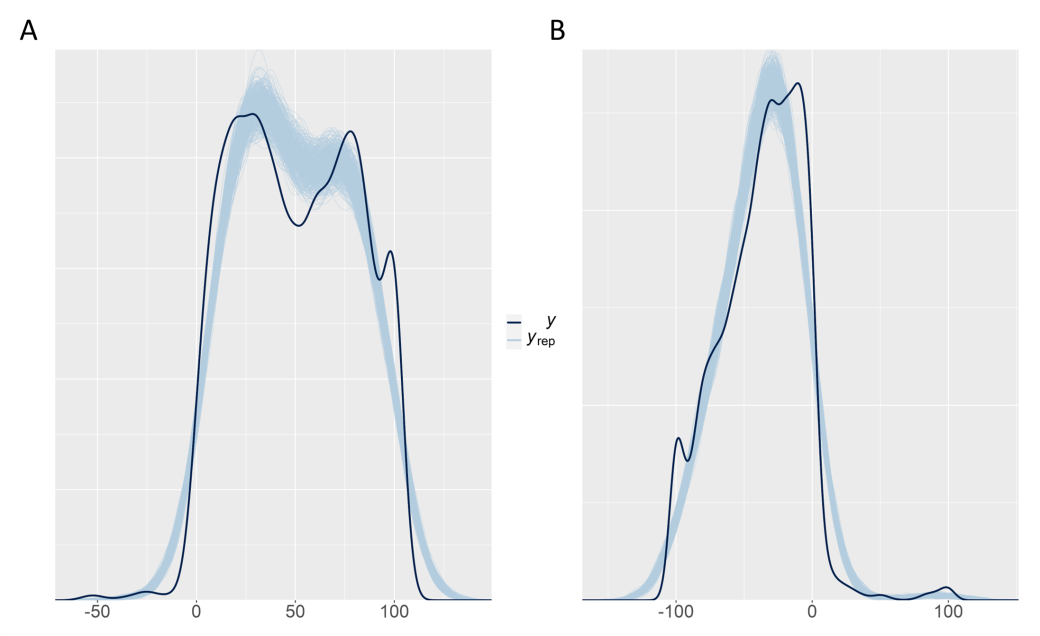


*Note.* Overlay of the density plots of 500 (randomly draw from the 16000 iterations) posterior predictions (y_rep_, colored in light blue) with the observed outcome (y, colored in black).

**Figure S3. Density plots indicating the posterior distribution of parameters within 95% HPDI (highest posterior density interval) for positive PTD (A) and negative PTD (B)**


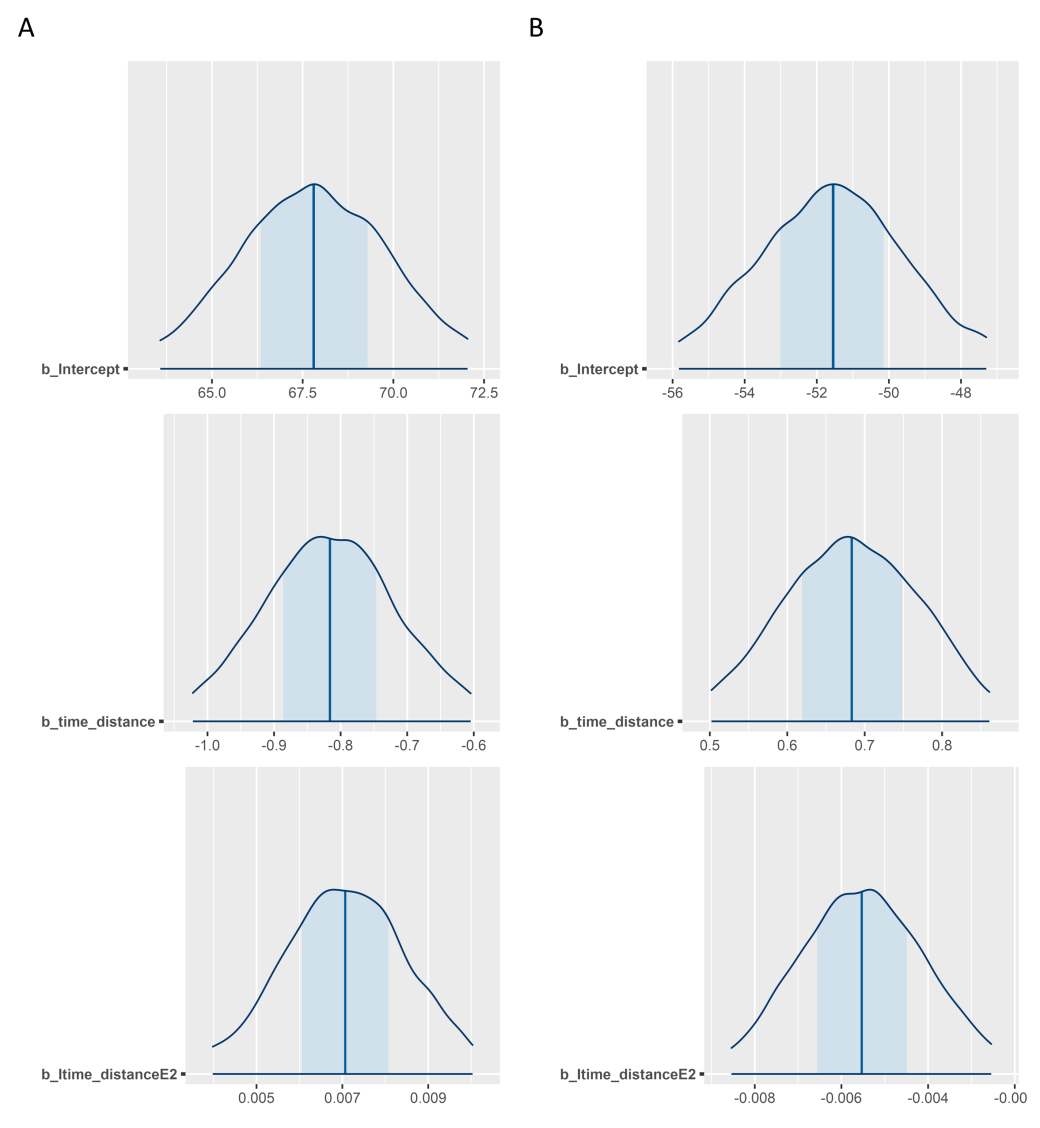


*Note.* The first row contains the density plots for the intercepts, while the second and third rows contain the density plots for linear and quadratic term of time distance. Of each density plot, the y-axis represents the density, while the x-axis shows the parameter value. The vertical line indicates the median, and the blue shade indicates 50% HPDI.

**Figure S4. Scatter plots indicating the association between PTD bias and multiple facets of mental health**


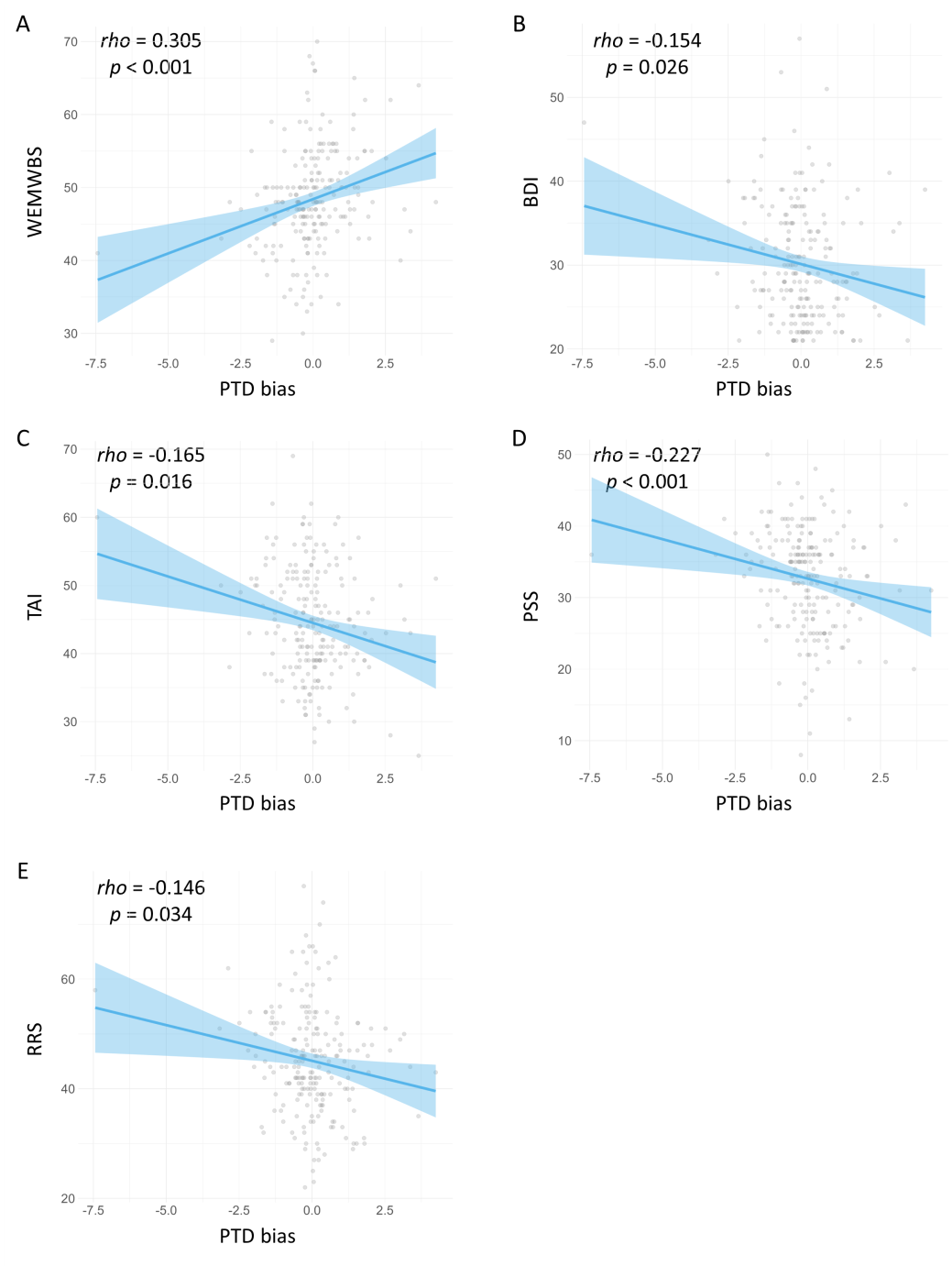


*Note.* X-axis is PTD bias, which is calculated as the difference between normalized positive AUC and normalized negative AUC. Y-axes are scores of multiple facets of mental health, including general mental well-being **(A)**, depression proneness **(B)**, anxiety proneness **(C)**, stress sensitivity **(D)**, rumination proneness **(E)**. Blue lines and shades represent the linear fit lines and their 95% confidence intervals. WEMWBS = Warwick-Edinburgh Mental Well-Being Scale. BDI = Beck's Depression Inventory. TAI = Trait Anxiety Inventory. PSS = Perceived Stress Scale. RSS = Ruminative Responses Scale.

| **Table S1. Results of multiple regression models indicating the effects of rates of positive and of negative AUC on multiple facets of mental health** | | | | | | | | | |
| --- | --- | --- | --- | --- | --- | --- | --- | --- | --- |
| Outcome Variable | Positive AUC | | | |  | Negative AUC | | | |
|  | *β* | 95% CI | *t* | *p* |  | *β* | 95% CI | *t* | *p* |
| WEMWBS | 0.284 | [0.147, 0.421] | 4.083 | <0.001 |  | -0.133 | [-0.27, 0.004] | -1.914 | 0.057 |
| BDI | -0.131 | [-0.272, 0.01] | -1.834 | 0.068 |  | 0.139 | [-0.002, 0.28] | 1.95 | 0.053 |
| TAI | -0.178 | [-0.318, -0.039] | -2.52 | 0.013 |  | 0.164 | [0.024, 0.303] | 2.314 | 0.022 |
| PSS | -0.162 | [-0.302, -0.021] | -2.274 | 0.024 |  | 0.149 | [0.009, 0.289] | 2.094 | 0.038 |
| RRS | -0.12 | [-0.261, 0.02] | -1.686 | 0.093 |  | 0.147 | [0.006, 0.287] | 2.052 | 0.042 |
| *Note:* *β* = standardized coefficients; CI = confidence interval. WEMWBS = Warwick-Edinburgh Mental Well-Being Scale. BDI = Beck's Depression Inventory. TAI = Trait Anxiety Inventory. PSS = Perceived Stress Scale. RSS = Ruminative Responses Scale. | | | | | | | | | |
